# Novel Findings of Anti-Filarial Drug Target and Structure-Based Virtual Screening for Drug Discovery

**DOI:** 10.3390/ijms19113579

**Published:** 2018-11-13

**Authors:** Tae-Woo Choi, Jeong Hoon Cho, Joohong Ahnn, Hyun-Ok Song

**Affiliations:** 1 Department of Life Science, Hanyang University, Seoul 04763, Korea; ctw1983@naver.com (T.-W.C.); joohong@hanyang.ac.kr (J.A.); 2 Department of Biology Education, College of Education, Chosun University, Gwangju 61452, Korea; renocho@chosun.ac.kr; 3 Department of Infection Biology, Wonkwang University School of Medicine, Iksan 54538, Korea

**Keywords:** filariasis, molecular modeling, virtual screening, anti-filarial drug, calumenin, itraconazole

## Abstract

Lymphatic filariasis and onchocerciasis caused by filarial nematodes are important diseases leading to considerable morbidity throughout tropical countries. Diethylcarbamazine (DEC), albendazole (ALB), and ivermectin (IVM) used in massive drug administration are not highly effective in killing the long-lived adult worms, and there is demand for the development of novel macrofilaricidal drugs affecting new molecular targets. A Ca^2+^ binding protein, calumenin, was identified as a novel and nematode-specific drug target for filariasis, due to its involvement in fertility and cuticle development in nematodes. As sterilizing and killing effects of the adult worms are considered to be ideal profiles of new drugs, calumenin could be an eligible drug target. Indeed, the *Caenorhabditis elegans* mutant model of calumenin exhibited enhanced drug acceptability to both microfilaricidal drugs (ALB and IVM) even at the adult stage, proving the roles of the nematode cuticle in efficient drug entry. Molecular modeling revealed that structural features of calumenin were only conserved among nematodes (*C. elegans*, *Brugia malayi*, and *Onchocerca volvulus*). Structural conservation and the specificity of nematode calumenins enabled the development of drugs with good target selectivity between parasites and human hosts. Structure-based virtual screening resulted in the discovery of itraconazole (ITC), an inhibitor of sterol biosynthesis, as a nematode calumenin-targeting ligand. The inhibitory potential of ITC was tested using a nematode mutant model of calumenin.

## 1. Introduction

Lymphatic filariasis and river blindness (onchocerciasis) are major neglected tropical diseases caused by infections with filarial nematodes. Lymphatic filariasis is transmitted by mosquito vectors (*Culex*, *Anopheles*, and *Aedes* spp.), and 974 million people in 54 countries worldwide remain threatened by the disease [1]. Three species of filarial parasites are responsible for lymphatic filariasis: *Wuchereria bancrofti*, *Brugia malayi*, and *Brugia timori* [2]. In the chronic condition, lymphatic filariasis leads to lymphedema (tissue swelling) or elephantiasis (skin/tissue thickening) of limbs. River blindness (onchocerciasis) caused by *Onchocerca volvulus* is transmitted by black flies (*Simulium* spp.) and it is endemic in 31 countries in sub-Saharan Africa, three countries in Latin America, and in Yemen [3]. Chronic infection results in itching and disfiguring skin lesions, and causes eye lesions which can develop into irreversible blindness.

Control and treatment strategies for these human filarial diseases include vector control and mass drug administration (MDA). Currently, diethylcarbamazine (DEC), albendazole (ALB), and ivermectin (IVM) are used in MDA programs at different regimens [4]. The combinational administration of two drugs among DEC, ALB, and IVM is recommended for lymphatic filaraisis, whereas IVM is the only drug that is available to treat onchocerciasis. In spite of their use in successful MDA programs, all of them have markedly shown limited macrofilaricidal (adult worm-killing) effects [5,6,7,8]. Furthermore, the issue of resistance to the drugs has become increasingly conspicuous [1,9], demanding the development of novel drugs affecting new molecular targets. It might be ideal if novel drugs fulfil several points: prominent macrofilaricidal effects or long-term sterilizing effects, no severe adverse reactions caused by worm killing, and safe usage for both children and pregnant/breastfeeding women [4].

Here, we propose a novel drug target, calumenin, which is a Ca^2+^ binding protein that is resident in the endoplasmic reticulum (ER). Calumenin belongs to the CREC (Cab45, reticulocalbin, ERC-55, and calumenin) family, and it is ubiquitously expressed in human cells, but highly expressed in the muscle and heart [10,11]. Similar expression has been observed in the free-living nematode, *Caenorhabditis elegans*. *C. elegans* calumenin is expressed in the pharynx, intestine, muscle, and hypodermis during development [12]. Both human and nematode calumenin possess a low Ca^2+^ affinity [10,12], and this is consistent with other Ca^2+^-binding proteins within the ER [13]. Although its differential expression was observed in malignant cells [14,15], the exact mechanisms for calumenin regulation are not yet known. The idea that calumenin could be a drug target was raised in our previous studies showing that calumenin mutant of *C. elegans* displayed reduced fertility as well as severe cuticle defects [12]. Reduced fertility of the mutant suggests that calumenin function in the regulation of breeding, and this is correlated with one of the ideal drug profiles, the sterilizing effect. The cuticle defect of the mutant is far more meaningful, because nematode cuticle largely contributes to efficient drug transfer from the outside [16,17,18,19,20]. Moreover, the nematode cuticle, a collagen-rich extra-cellular matrix, is crucial for development and survival. Therefore, enzymes and chaperones involved in cuticle development have been suggested as potential drug targets for parasitic nematodes [21,22].

Recently, remarkable advances in computer algorithms for predicting protein structures have enabled biologists to predict three-dimensional (3D) structures of their proteins of interest, starting from an amino acid sequence [23]. Then, the predicted protein models can be applied to computational ligand-binding studies as well as virtual compound screening. Besides, repositioning of FDA-approved drugs for new indications has gained significant attention in this kind of screening, due to its time and cost effectiveness in clinical use [24]. Here, we report the results of the structural modeling of nematode calumenins (*C. elegans*, *B. malayi*, and *O. volvulus*) and human calumenin. We found that the structural features of calumenin are well conserved in both free-living and filarial nematodes, representing the potential of calumenin as a nematode-specific target. We further tried drug repositioning through virtual screening with their predicted protein models. Finally, itraconazole (ITC), a triazole anti-fungal agent, was screened as a nematode calumenin-specific compound. The inhibitory potential of the drug was examined using the *C. elegans* mutant model of calumenin.

## 2. Results

### 2.1. Functional Loss of Calumenin-Enhanced Sensitivity to Known Anti-Filarial Drugs

**Figure 1:**
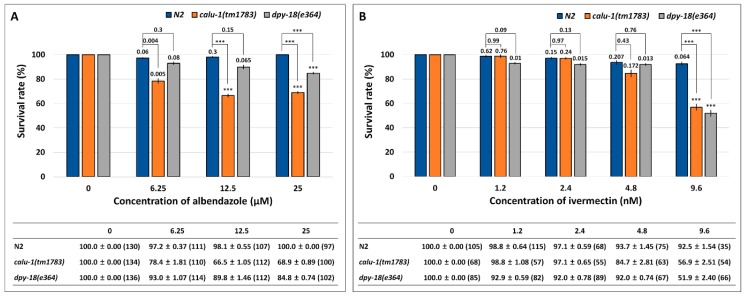
Enhanced sensitivity of the *calu-1*(*tm1783*) mutant against (**A**) albendazole (ALB) and (**B**) ivermectin (IVM). A survival test was conducted on food-supplemented nematode growth media (NGM) containing various concentrations of drugs. Survival was measured in 24 h intervals for three days after drug treatment (See Method details). Mean survival rate (%) ± SEM (*n* number) was shown in figure and table at three days post-exposure of ALB (**A**) and at two days post-exposure of IVM (**B**). Survival rate was normalized by control (untreated). The *p* values were evaluated by *t*-test on both untreated vs. treated and wild type vs. mutant. *** *p* < 0.001.

The *C. elegans* mutant of calumenin (*calu-1*(*tm1783*)) has been shown to display severe cuticle defects [12]. As the nematode cuticle plays a role in drug entry, it is reasonable to assume that the calumenin mutant may exhibit enhanced sensitivity to drugs. To test this possibility, the *calu-1*(*tm1783*) mutant was treated with two anti-filarial drugs, ALB and IVM, which are mainly used for the control and treatment of filariasis. Survival rate was measured up to three days after drug treatment, and the result was compared to the wild type (N2). The *dpy-18*(*e364*) mutant was included in the test, because *dpy-18* encodes an α subunit of prolyl 4-hydroxylase that is crucial for collagen biosynthesis [25,26]. As a result, the wild type (N2) did not show statistically significant differences between untreated and drug-treated groups in the case of both drugs (Figure 1). However, as shown in Figure 1A, the *calu-1*(*tm1783*) mutant showed statistically significant differences between the untreated group and the treated group at all concentrations of ALB (*p* = 0.005, *p* < 0.001, and *p* < 0.001 at each drug concentration), and the sensitivity was significantly higher than the wild type (N2) (*p* = 0.004, *p* < 0.001, and *p* < 0.001 at each drug concentration), suggesting its hypersensitivity to ALB. The *dpy-18*(*e364*) mutant was also more sensitive to ALB than the wild type (N2), but it was less sensitive than the *calu-1*(*tm1783*) mutant. Statistical significance was observed only in the 25 μM ALB conditions (both untreated vs. treated and wild type vs. mutant) (Figure 1A). The *calu-1*(*tm1783*) mutant was also more sensitive to IVM, compared to the wild type (N2) but statistical significance was only observed in the 9.6 nM IVM condition (both untreated vs. treated and wild type vs. mutant) (Figure 1B). In contrast, the *dpy-18*(*e364*) mutant showed a statistically sensitive response to the drug at all concentrations tested (*p* = 0.01, *p* = 0.015, *p* = 0.013, and *p* < 0.001 at each drug concentration) (Figure 1B). However, statistical difference between the wild type (N2), and the mutant was only observed in the 9.6 nM IVM condition as in the *calu-1*(*tm1783*) mutant. Taken together, these results suggest that the malfunction of the cuticle could be directed towards efficient drug delivery in worms, and therefore, calumenin, a crucial factor for normal cuticle development, could serve as a novel drug target.

### 2.2. Calumenin Proteins are Highly Conserved between Free-Living and Filarial Nematodes

**Figure 2:**
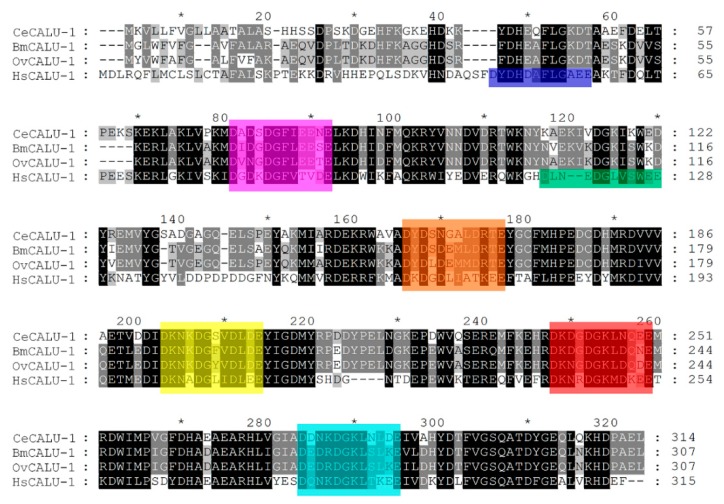
Pairwise and global alignments of *C. elegans* calumenin (CeCALU-1), *B. malayi* calumenin (BmCALU-1), *O. volvulus* calumenin (OvCALU-1), and *H. sapiens* calumenin (HsCALU-1). Conserved Ca^2+^ binding EF hand motifs among all species are shown in magenta, orange, yellow, red, and cyan, in order. Extra EF hand motifs of human calumenin are shown in blue for the first, and green for the third. The multiple sequence alignment was performed with Clustal X. Alignment data were further visualized and analyzed by GeneDoc. Shading is according to alignment consensus as given by GeneDoc (black, 100%; dark gray, 80%; light gray, 60%). * indicates a marker for counting 10 amino acids. GenBank accession no. AAF34189.1 (CeCALU-1); AAB97725.1 (HsCALU-1).

Calumenin is well conserved from worm to human [11]. The *C. elegans* calumenin protein shares a 45% identity with its human counterpart [12]. A calumenin homolog has been identified in both filarial nematodes, *B. malayi* and *O. volvulus* (Bm5089 and OVOC5386, respectively) [27,28]. *C. elegans* calumenin (CeCALU-1) showed a 72% identity in amino acid sequences with ones of two filarial nematodes (Figure 2). *B. malayi* calumenin (BmCALU-1) and *O. volvulus* calumenin (OvCALU-1) showed 43% and 44% identity in amino acid sequences with human calumenin (HsCALU-1), respectively. Filarial calumenins are highly conserved each other (91% identity in amino acid sequences). They contain five putative EF hand motifs, which are well conserved among three nematode calumenins, whereas HsCALU-1 contains seven of them. Both BmCALU-1 and OvCALU-1 have the C-terminal ER (endoplasmic reticulum) retention signal, PAEL (Proline-Alanine-Glutamic acid-Leucine), as does CeCALU-1.

### 2.3. Molecular Modeling of the Calumenin Protein Structure

**Figure 3:**
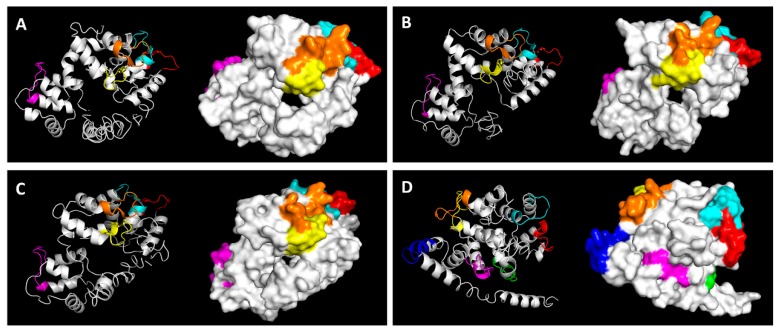
Predicted three-dimensional (3D) structure of (**A**) *C. elegans* calumenin (CeCALU-1), (**B**) *B. malayi* calumenin (BmCALU-1), (**C**) *O. volvulus* calumenin (OvCALU-1), and (**D**) *H. sapiens* calumenin (HsCALU-1) All structures are presented in two viewing modes (cartoon and surface) by PyMOL. Ca^2+^ binding EF hand motifs are colored as in Figure 2.

Homology modeling of nematode and human calumenins was performed through the I-TASSER server, an integrated platform for automated protein structure and function prediction based on the sequence-to-structure-to-function paradigm [29,30,31,32]. Based on the amino acid sequences of calumenins, template proteins were firstly identified from a solved structure database that had a similar structure or motifs. The top 10 template hits were then selected for the structural assembly (Table S1). The top five models were predicted from each calumenin sequence, and the best model was finally selected for each of them. The confidence of each protein model was quantitatively measured by the confidence score (C-score), which is calculated based on the significance of template alignments and the convergence parameters of the structure assembly simulation. The template modeling score (TM-score) and the root-mean-square deviation (RMSD) were also estimated to predict the quality of the modeling prediction based on the C-score and the protein length, following the correlation observed between these qualities. The finalized best models of nematode and human calumenins were visualized by PyMOL, a molecular visualization system on an open source foundation (Figure 3) [33]. The C-score, the estimated TM-score, and the estimated RMSD for each model were shown in Table 1.

**Figure 4:**
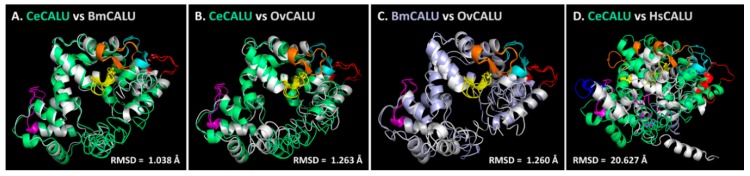
Structural alignment among calumenins. (**A**) *C. elegans* calumenin (CeCALU-1) vs. *B. malayi* calumenin (BmCALU-1). (**B**) *C. elegans* calumenin (CeCALU-1) vs. *O. volvulus* calumenin (OvCALU-1). (**C**) *B. malayi* calumenin (BmCALU-1) vs. *O. volvulus* calumenin (OvCALU-1). (**D**) *C. elegans* calumenin (CeCALU-1) vs. *H. sapiens* calumenin (HsCALU-1). CeCALU-1 is shown in lime green. BmCALU-1 was shown in gray in (**A**) and light violet in (**C**). OvCALU-1 and HsCALU-1 are shown in gray. EF hand motifs are colored as in Figure 2.

As shown in Figure 3, the overall structures of three nematode calumenins seemed to be similar, whereas the predicted structure of human calumenin looked different. Four of the five EF hand motifs (shown in orange, yellow, red, and cyan in Figure 3) were closely located in all three nematode calumenin models, but the situation was different in the human calumenin model. Four of the seven EF hand motifs were closely located in pairs (shown in orange and yellow for one, and red and cyan for the other in Figure 3), and the remaining three were far apart in the model. The HsCALU-1 model showed a long helical tail in the N-terminal region. Thus, the structure model of calumenin was aligned to one another to calculate the RMSD, which is the measure of the average distance between atoms (Figure 4).

CeCALU-1 model showed high structural similarity with BmCALU-1 (RMSD = 1.038 Å) and the OvCALU-1 model (RMSD = 1.263 Å) (Figure 4A,B). The BmCALU-1 and OvCALU-1 model also displayed high structural similarities with a RMSD value of 1.260 Å (Figure 4C). However, the RMSD between the CeCALU-1 and HsCALU-1 model was considerably higher (20.627 Å) (Figure 4D), suggesting the significant difference between the structures. The same pattern was observed in the structure alignment between BmCALU-1 and HsCALU-1 (RMSD = 19.877 Å) or between OvCALU-1 and HsCALU-1 (RMSD = 19.918 Å). These results suggest that nematode calumenins might share well-conserved structural features, but not with human calumenin. This strongly suggests that calumenin could be a nematode-specific drug target.

### 2.4. Virtual Screening of Approved Drugs by Molecular Docking

In order to screen ligands targeting nematode calumenins, molecular docking was performed using the predicted 3D structures of calumenin. The Zdd library of 1701 compounds representing commercially available approved drugs was screened using RyRx, an open source virtual screening software [34]. The same set of ligands was independently screened with the individual 3D structure of BmCALU-1, CeCALU-1, OvCALU-1, and HsCALU-1 to find compounds that specifically bind to nematode calumenins (BmCALU-1, CeCALU-1, and OvCALU-1) but not to human calumenin (HsCALU-1). Screened chemicals and corresponding names were listed in the order of the binding affinities between the BmCALU-1 model and the ligand (Table 2). The ranking of binding affinities between the same ligand and one of the other CALU-1 models (CeCALU-1 or OvCALU-1 or HsCALU-1 model) was described either in the Table 2.

Some of them showed strong binding affinity to both nematode and human calumenins, showing high rankings in all screenings (e.g., nos. 1, 2, 3, and 9). On the other hand, some of them displayed strong binding affinity only to BmCALU-1 model (e.g., nos. 4, 5, 7, 14, and 21). In order to screen potential drug candidates, chemicals having high affinity only to nematode calumenins (BmCALU-1, CeCALU-1, and OvCALU-1) were specifically focused on (nos. 8, 19, 24, 34, and 44). Those chemicals showed relatively low binding affinity to human calumenin, providing nematode specificity. Among them, no. 19, itraconazole (ZINC03830976), was finally selected to test its potential as a targeting ligand of nematode calumenin.

### 2.5. The calu-1(tm1783) Mutant Exhibited Resistance to Itraconazole

**Figure 5:**
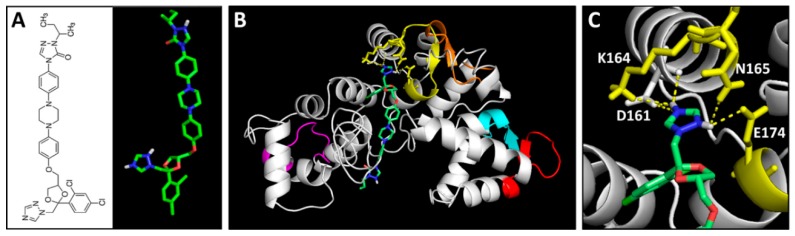
Predicted binding mode of itraconazole (ITC) to BmCALU-1. (**A**) Chemical and 3D structures of ITC. Overall docking mode (**B**) and detailed magnified image (**C**) of ITC to BmCALU-1 were resolved by PyMOL. Interaction was analyzed within a 3 Å distance. EF hand motifs are colored as in Figure 2. ITC carbon atoms are colored green, oxygen red, and nitrogen blue.

The chemical and 3D structures of itraconazole (ITC) are shown in Figure 5A. The predicted binding mode of ITC and BmCALU-1 was further resolved and visualized by PyMOL (Figure 5B,C). It was found that ITC docks with the binding pocket of BmCALU-1, and it interacts with three residues (Lys164, Asn165, and Glu174) located in the third EF hand motif (shown in yellow) as well as one residue (Asp161) that is adjacent to the third EF hand motif.

**Figure 6:**
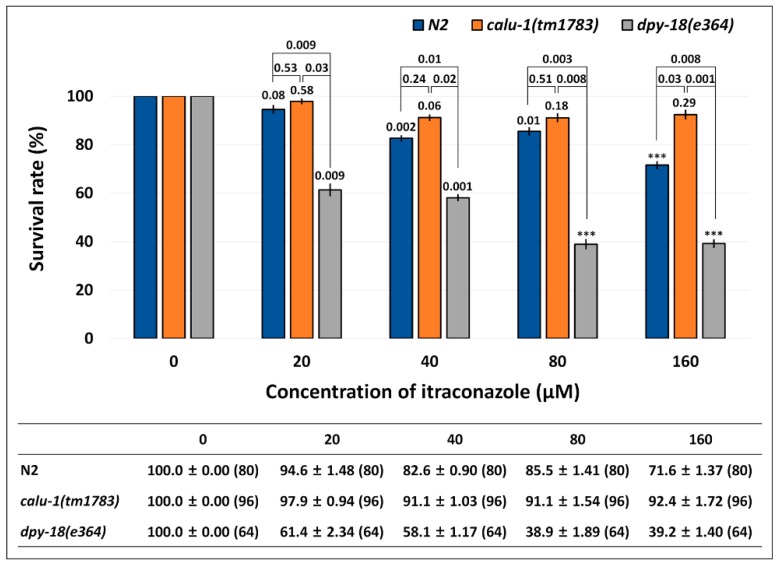
Resistance of *calu-1*(*tm1783*) mutant against ITC. A survival test was conducted in food-supplemented M9 buffer containing a various concentration of the drug. Survival was measured in 24 h intervals for three days after drug treatment (See Method details). The mean survival rate (%) ± SEM (*n* number) are shown in the figure and table at three days post-exposure. Survival rate was normalized by the control (untreated). The *p* values were evaluated by *t*-test on both untreated vs. treated and wild type vs. mutant. *** *p* < 0.001.

In order to test whether ITC indeed targets nematode calumenin, a survival test was conducted using wild type (N2), *calu-1*(*tm1783*) and *dpy-18*(*e364*) mutants in an assay solution containing a various concentration of ITC (0, 20, 40, 80, and 160 μM). We assume that if ITC interacts and inhibits nematode calumenin, the *calu-1*(*tm1783*) mutant may be resistant to the drug. This may be because the mutant does not have the normal target (calumenin) that ITC can bind to and inhibit. On the other hand, if ITC is the only inhibitor for calumenin, we can expect that ITC will not affect the other mutations, such as *dpy-18*(*e364*), which is found to be sensitive to test drugs (ALB and IVM) due to its cuticle defect. As shown in Figure 6, the wild type (N2) appeared to be significantly sensitive to ITC from the 40 μM drug condition (*p* = 0.002). The *dpy-18*(*e364*) mutant was also significantly sensitive to the drug at all concentrations tested (*p* = 0.009, *p* = 0.001, *p* < 0.001, and *p* < 0.001 at each drug concentration). Statistically significant differences were observed in all concentrations between wild type (N2) and the mutant (*p* = 0.009, *p* = 0.01, *p* = 0.003, and *p* = 0.008 at each drug concentration), suggesting that the *dpy-18*(*e364*) mutant, like ALB and IVM, is hypersensitive to ITC.

Interestingly, the *calu-1*(*tm1783*) mutant was resistant to ITC at all of the concentrations tested. No statistical differences were observed between the untreated group and the treated group of the mutant (*p* = 0.58, *p* = 0.06, *p* = 0.18, and *p* = 0.29 at each drug concentration). The *calu-1*(*tm1783*) mutant showed statistically different responses to ITC compared to the wild type (N2), only in the 160 μM ITC condition (*p* = 0.03). However, compared to the *dpy-18*(*e364*) mutant, it showed statistically significant differences at all concentrations of the drug (*p* = 0.03, *p* = 0.02, *p* = 0.008, and *p* = 0.001 at each drug concentration). These results suggest that only the *calu-1*(*tm1783*) mutant exhibited the resistant phenotype to ITC, indicating that ITC indeed selectively targets nematode calumenin.

## 3. Discussion

Calumenin, a member of the CREC family, is a Ca^2+^ binding protein that contains EF hand motifs and a C-terminal ER retention signal [10,35,36]. In a previous study, we have shown that *C. elegans* calumenin is required for regulating fertility, locomotion, and body size [12]. Most of all, abnormal locomotion and the reduced body size of *calu-1*(*tm1783*) mutant is highly correlated with its severe cuticle defect. As the nematode cuticle is crucial for its development and survival, it has been suggested as a potential drug target for parasitic nematodes [21,22]. In particular, a collagen modifying enzyme, prolyl 4-hydroxylase, essential for collagen biosynthesis, has received attention in filarial nematodes, *B. malayi* and *O. volvulus* [37,38]. In this study, we suggest calumenin as a novel and nematode-specific drug target for filariasis for several reasons. First, the loss of calumenin function enhanced drug acceptability, even at the adult stage, with an expected macrofilaricidal effect of the drugs, if developed (Figure 1). Secondly, the functional loss of calumenin reduced fertility [12], with an expected sterilizing effect of the drugs, if developed. Finally, molecular modeling showed significantly distinct structures of nematode calumenins from the human counterpart, providing target selectivity between the parasite and the human host (Figure 2, Figure 3 and Figure 4). It was found that amino acid sequences and predicted structures were well conserved in both free-living (*C. elegans*) and parasitic filarial nematodes (*B. malayi* and *O. volvulus*). Thus, selective calumenin-targeting drugs might be effective to both lymphatic filariasis and river blindness (onchocerciasis).

The 3D structures of nematode and human calumenins were predicted based on sequence and structure homology using I-TASSER [29,30,31,32]. The quality of predicted protein models was evaluated with several parameters, including the C-score, the estimated TM-score, and the estimated RMSD. Based on I-TASSER statistics [29,30,31,32], the C-score, a confidence score, typically ranges from −5 to 2, and a C-score > −1.5 indicates a model of correct global topology. The RMSD and the TM-score [39] are scales to evaluate the structural similarity between two structures by measuring the distance between the predicted model and the native structures. In traditional comparative modeling (CM) using close homologous templates, a high resolution model is generated with a RMSD of 1−2 Å [40]. Medium-resolution models, which are typically generated by threading and CM from distantly homologous templates, are roughly in the RMSD range of 2−5 Å, with errors mainly in the loop region [30,41]. However, the RMSD is not meaningful for measuring the modeling quality in the case of lower accuracy models, because the RMSD is an average distance of all residue pairs in two structures, and thus, a local error occurring mainly in tails or loops can bring about a big RMSD value, although the core region of the model is correct. Therefore, the quality of prediction was evaluated with the TM-score. In the TM-score, a larger distance of errors are scored with a lower weight than a smaller distance, making the score more sensitive [39]. In addition, the correlation coefficient for the C-score and the TM-score (0.91) has been reported to be much higher than that of the C-score and the RMSD (0.75) in the server’s test [29,30]. By definition, a TM-score ≤ 0.17 means a random similarity, and a TM-score > 0.5 indicates a model of correct topology. Considering the various parameters, the quality of predicted calumenin structures seem to be relatively less reliable, except BmCALU-1 (Table 1). Thus, the BmCALU-1 model was subsequently used to analyze the docking mode of itraconazole (ITC) (Figure 5). Despite the limit in protein models, the structural similarity was significantly high among nematode calumenin models (Figure 4). Thus, a chemical library was screened with all predicted calumenin models to discover common and selective ligands that target only nematode calumenins (Table 2).

We screened the library containing drugs that have been approved for use in humans somewhere in the world, and that are commercially available as pure compounds. This is because the repurposing of drugs is less risky and a faster way to discover drugs with already known safety and pharmacokinetics profiles [42,43]. Actually, drug repositioning has recently discovered valuable drug candidates for parasitic nematodes such as hookworms [44] and endosymbionts of filarial nematodes [45]. Independent virtual screening in this study resulted in several drug candidates that might specifically react with nematode calumenins (ZINC03830631, daunorubicin hydrochloride; ZINC03830924, idarubicin; ZINC03830976 and ZINC04097344, ITC; ZINC04214700, paliperidone in Table 2).

Daunorubicin hydrochloride (ZINC03830631) is known to be a potent anti-cancer agent, which inhibits DNA and RNA synthesis by intercalating double-stranded DNA [46]. It also acts as an inhibitor for DNA topoisomerase II, thereby inhibiting DNA replication, repair, and RNA/protein synthesis [47]. Idarubicin (ZINC03830924) is a semisynthetic 4-demethoxy analog of daunorubicin hydrochloride, exhibiting mostly the same pharmacological profiles [48,49,50]. Paliperidone (ZINC04214700) is a second-generation antipsychotic agent, an active metabolite of risperidone, which is used for the treatment of schizophrenia by functioning as a dopamine D_2_ receptor antagonist and a serotonin 5-HT_2_ receptor antagonist [51,52,53]. These drugs were excluded in further studies due to relatively their lower target selectivities between parasites and hosts.

Instead, ITC was paid more attention because three analogs of ITC (ZINC03830974, ZINC03830976, and ZINC04097344), were all screened to have higher affinity to nematode calumenins. It has been known to inhibit a lanosterol 14-α-demethylase, an enzyme to convert lanosterol to ergosterol, which is an essential component of the fungal cell membrane, thus presenting anti-fungal activity [54,55,56]. The inhibitory potential of ITC to nematode calumenin was subsequently tested with the *C. elegans* mutant of calumenin as the representative model organism of filarial nematodes. A survival test was conducted in a liquid assay format to improve the drug solubility. Interestingly, the results showed a statistically significant resistance of *calu-1*(*tm1783*) mutant to ITC compared to the wild type (N2) (*p* = 0.03 at 160 μM). By contrast, the *dpy-18*(*e364*) mutant exhibited more sensitive responses to ITC, compared to the wild type (N2) at all tested concentrations (*p* = 0.009, *p* = 0.01, *p* = 0.003, and *p* = 0.008 at each drug concentration). Given the fact that both the *calu-1*(*tm1783*) mutant and the *dpy-18*(*e364*) mutant were found to be hypersensitive to the test drugs (ALB and IVM) due to the increased penetration of the drug through their deformed cuticle, the different responses of two mutants to ITC prove that ITC actually targets only calumenin. However, the tolerant phenotypes of the *calu-1*(*tm1783*) mutant against the drug were not dramatic compared to the wild type (N2) organism. This might have originated from the nature of the *calu-1*(*tm1783*) mutant. The *calu-1*(*tm1783*) is a loss-of-function mutant, which contains a deletion in the 5′ untranslated region (5’-UTR), the first two exons, and the splice donor site of the second intron, resulting in an N-terminal truncated protein [12]. The deletion does not affect any EF-hand motifs of calumenin. Considering that ITC interacts with residues that are mostly located in the third EF hand motif of calumenin (BmCALU-1; Figure 5), the binding of ITC could not be critically inhibited in the *calu-1*(*tm1783*) mutant. This is further supported by the binding mode of ITC being somehow conserved in CeCALU-1 model, in spite of its limited reliability. ITC was predicted to interact with two residues (Lys195 and Asn196) located at the same position in the third EF hand motif of CeCALU-1 within a 3.5 Å distance. We also cannot ignore that the effective concentration of ITC was found to be much higher than the known anti-filarial drugs tested (ALB and IVM). This suggests that the drug has valid potential for killing adult worms by targeting calumenin, but this has to be further optimized for the best efficacy at the same time.

Sterols are crucial for various functions in most eukaryotic cells, and thus, most organisms including yeast, plants, insects, and mammals have complex biosynthetic pathways for sterol [57]. By contrast, both free-living and parasitic nematodes cannot synthesize sterols de novo, and thus, they do not have any homologs that are responsible for a lanosterol 14-α-demethylase, the original target of ITC [58,59]. The screen conducted in this study discovered ITC as a potential binding ligand of nematode calumenin. Interestingly, the posture of ITC binding to nematode calumenin appears to be similar to that of the *Saccharomyces cerevisiae* lanosterol 14-α-demethylase and ITC [60]. The triazole group of ITC makes bonds with several residues of nematode calumenin or the heme iron of *S. cerevisiae* lanosterol 14-α-demethylase, and the long tail of ITC fills the entry channels of both molecules (Figure S1). This actually suggests possible bindings between ITC and the nematode calumenin.

This study has discovered a novel and nematode-specific drug target, calumenin, which is essential for the viability of the nematode. Calumenin seems to be involved in normal cuticle development and fertility, although its exact roles and mechanisms are as-yet uncovered. Cuticle collagen must be newly synthesized between molting periods in order to allow the nematode to grow and to expand its body size. This is crucial for normal growth and survival in both free-living and parasitic nematodes. Cuticle collagen biosynthesis is a complex multi-step process including post-translational modification, folding, and processing. Calumenin might be one of numerous enzymes and chaperones involved in this collagen biosynthesis. As calumenin localizes at the lumen of ER and prolyl 4-hydroxylation occurs in the site, we assume that calumenin may have roles in this first important co-translational modification of procollagen. Along with this hypothesis, we found that the *dpy-18*(*e364*);*calu-1*(*tm1783*) double mutant is larval-lethal, suggesting that calumenin may have indispensable roles with prolyl 4-hydroxylase in cuticle collagen biogenesis. However, further studies are required to solve the precise function of calumenin in this biological process. Calumenin is known to be expressed in the vulva muscle, mediating egg-laying, but it is not yet clear whether the reduced fertility of *calu-1*(*tm1783*) mutant is caused by a muscular defect or by a neuronal defect, or by both. Thus, further studies are necessary to reveal the roles of calumenin in nematode fertility. Meanwhile, the precise 3D structure of calumenin must be resolved by X-ray crystallography to develop calumenin structure-specific inhibitors as anti-filarial drug candidates.

## 4. Materials and Methods

### 4.1. Chemicals

All chemicals were purchased from Sigma-Aldrich (St. Louis, MO, USA): albendazole (A4673), ivermectin (I8898), itraconazole (I6657), and dimethyl sulfoxide (DMSO, D4540).

### 4.2. C. elegans Strains and Cultivation

The following strains were obtained from the *Caenorhabditis* Genetics Center (CGC, available online: https://cgc.umn.edu): Bristol N2, CB364 *dpy-18*(*e364*) III. The *calu-1*(*tm1783*) X mutant was obtained from the National BioResource Project, Tokyo, Japan. Standard methods for worm breeding and handling were used, as previously described [61].

### 4.3. Drug Sensitivity Assay

The worms of forth larval stage (L4 larvae) of each strain were transferred to nematode growth media (NGM) seeded with *Escherichia coli* OP50 strain as food sources and allowed to grow 24 h at 20 °C. The resultant 1-day-old adults were then examined for drug sensitivity. All chemicals were dissolved with DMSO. The maximum concentration of DMSO was below 1%. Survival was checked at 24 h intervals for three days. Worms were considered dead when they showed no response to touch or tapping of the plate, as well as when they lacked pharyngeal pumping. The worms that crawled off the plate were excluded from the counting. The measurement was repeated at least three times. Solid plate killing assay for ALB and IVM: NGM was prepared with a various concentration of drugs and seeded with OP50. Ten to 15 1-day-old adults were transferred to drug plates, and cultivated at 20 °C. Liquid killing assay for ITC: Assay solution was prepared with 80% (*v*/*v*) M9 buffer (22 mM KH_2_PO_4_, 42.3 mM Na_2_HPO_4_, and 85.6 mM NaCl) and 20% (*v*/*v*) OP50-cultured solution with various concentrations of drug. One hundred microliters (100 μL) of assay solution were distributed to a single well of a 96-well culture plate (Cat#32096, SPL Life Sciences, Pocheon, Korea). Single animals were transferred to each well and incubated at 20 °C.

### 4.4. Homology Modeling of Calumenin Structure

Amino acid sequences of CeCALU-1 (GenBank accession no. AAF34189.1), BmCALU-1 (Bm5089), OvCALU-1 (OVOC5386), and HsCALU-1 (GenBank accession no. AAB97725.1) were used to predict the calumenin structure with I-TASSER (available online: http://zhanglab.ccmb.med.umich.edu/I-TASSER) [29,30,31,32]. Briefly, by a variety of sequence-based and structure-based scores, the top 10 templates were identified by LOMETS (Available online: https://zhanglab.ccmb.med.umich.edu/LOMETS), a meta-server threading approach containing multiple threading programs, from the PDB library. Then, I-TASSER combines different aligned threaded templates to build a model. The template structures are assembled to make structural conformations of well-aligned regions, and the unaligned regions are modeled by ab initio simulations in I-TASSER. The fragment assembly is performed by a modified replica-exchanged Monte Carlo simulation, and the conformations generated in low-temperature replicas are clustered by SPICKER program (Available online: https://zhanglab.ccmb.med.umich.edu/SPICKER) in the server. The fragment assembly simulation is then performed again, starting from the selected cluster centroids, to refine their global topology. The structural conformations generated during this second simulations are clustered again to generate the final structural model. Each predicted calumenin model was visualized by PyMOL (The PyMOL Molecular Graphics System, Version 1.8 Schrödinger, Limited liability company; available online: https://www.pymol.org). Structural similarity among calumenin models were examined with PyMOL by calculating root-mean-square deviation (RMSD).

### 4.5. Virtual Screening of Drugs by Molecular Docking

Predicted 3D models of calumenins were used to screen the library of compounds by molecular docking with RyRx/AutoDock Vina (Version 0.8; available online: http://pyrx.sourceforge.net). The subset of molecule that contains 1701 commercially available approved drugs (Zdd) was obtained from the ZINC database (Version 12; available online: http://zinc.docking.org) [62]. The ligands were retrieved in Open Babel in PyRx, and energy-minimized by 200 steps using a universal force field (uff) before docking to remove clashes among atoms of the ligand, and to make a reasonable starting pose. The ligands were then converted to the AutoDock ligand format (pdbqt: protein data bank, partial charge (q), and atom type (t)) as previously described [34]. A grid box was generated with the maximized size for each protein model (pdbqt) by using AutoGrid embedded in PyRx/AutoDock Vina. After virtual screening was completed, PyRx automatically proceeded to the Analyze Results page. The predicted binding mode and corresponding binding affinity (binding energy, kcal/mol) are shown in this page. The negative value for the binding affinity indicates that the ligand is predicted to bind to a target macromolecule. Finally, binding affinities (kcal/mol) between each calumenin model and ligands were predicted and compared. The detailed binding mode of ITC to calumenin was predicted to within 3 Å distance, and visualized by PyMOL.

## Figures and Tables

**Table 1:** Quality parameters of three-dimensional (3D) model prediction.

Parameter	Predicted Protein Model			
	CeCALU-1	BmCALU-1	OvCALU-1	HsCALU-1
C-score	−2.95	−1.50	−2.81	−1.98
Estimated TM-score	0.38 ± 0.13	0.53 ± 0.15	0.39 ± 0.13	0.48 ± 0.15
Estimated RMSD	13.4 ± 4.1 Å	9.5 ± 4.6 Å	12.9 ± 4.2 Å	10.9 ± 4.6 Å

**Table 2:** List of compounds screened by molecular docking.

No *	ZINC ID	Score Ranking (Binding Affinity, kcal/mol)				Chemical Name
		BmCALU-1	CeCALU-1	OvCALU-1	HsCALU-1	
1	ZINC06716957	1 (−11.2)	9 (−9.7)	8 (−10.7)	5 (−9.4)	Nilotinib
2	ZINC52955754	2 (−11)	2 (−10.8)	1 (−11.9)	1 (−9.8)	Ergotamine
3	ZINC14880002	3 (−10.9)	5 (−10.2)	19 (−10.4)	2 (−9.7)	Ergoloid
4 **	ZINC02033588	4 (−10.7)	142 (−8.5)	86 (−9.4)	273 (−7.9)	Silybin
5 **	ZINC03831449	5 (−10.5)	77 (−8.7)	60 (−9.7)	105 (−8.3)	Silybin
6	ZINC01530886	6 (−10.3)	16 (−9.3)	15 (−10.6)	24 (−8.7)	Telmisartan
7 **	ZINC14261579	7 (−10.2)	177 (−8.4)	60 (−9.7)	105 (−8.3)	Ciclesonide
8 **	ZINC04097344	8 (−10.1)	31 (−9)	24 (−10.3)	78 (−8.4)	Itraconazole
9	ZINC12503187	8 (−10.1)	3 (−10.3)	3 (−11)	6 (−9.1)	Conivaptan
10 **	ZINC03915154	8 (−10.1)	11 (−9.4)	106 (−9.3)	34 (−8.6)	Ciclesonide
11 **	ZINC03913937	11 (−10)	23 (−9.2)	201 (−8.8)	128 (−8.2)	Piperacillin sodium
12 **	ZINC11592732	11 (−10)	108 (−8.6)	5 (−10.8)	128 (−8.2)	Piperacillin sodium
13	ZINC03831506	11 (−10)	10 (−9.6)	51 (−9.8)	24 (−8.7)	[9,10-dihydroxy-3-(2-thienyl)-2,4,7-trioxabicyclo[4.4.0] dec-8-yl]oxy-(4-hydroxy-3,5-dimethoxy-phenyl
14	ZINC18324776	14 (−9.9)	450 (−7.8)	154 (−9)	105 (−8.3)	Vardenafil
15	ZINC03932831	14 (−9.9)	3 (−10.3)	24 (−10.3)	4 (−9.5)	Dutasteride
16	ZINC19796084	14 (−9.9)	77 (−8.7)	46 (−9.9)	24 (−8.7)	Pimozide
17	ZINC19632618	14 (−9.9)	10 (−9.6)	32 (−10.1)	8 (−8.9)	Imatinib
18	ZINC03830261	18 (−9.8)	52 (−8.8)	282 (−8.5)	748 (−7.1)	Azlocillin sodium
19 **	ZINC03830976	18 (−9.8)	8 (−9.8)	8 (−10.7)	168 (−8.1)	Itraconazole
20	ZINC03927200	18 (−9.8)	1 (−11.1)	121 (−9.2)	24 (−8.7)	Drospirenone
21	ZINC18324776	18 (−9.8)	450 (−7.8)	154 (−9)	128 (−8.2)	Vardenafil
22	ZINC18098320	18 (−9.8)	40 (−8.9)	79 (−9.5)	433 (−7.6)	Chlorhexidine
23	ZINC04026555	24 (−9.7)	108 (−8.6)	37 (−10)	34 (−8.6)	Estradiol benzoate
24	ZINC03830924	24 (−9.7)	23 (−9.2)	8 (−10.7)	128 (−8.2)	Idarubicin
25	ZINC13298436	24 (−9.7)	77 (−8.7)	60 (−9.7)	34 (−8.6)	Irbesartan
26	ZINC03977978	24 (−9.7)	40 (−8.9)	219 (−8.7)	34 (−8.6)	Fluocinonide
27	ZINC03914596	24 (−9.7)	7 (−9.9)	2 (−11.2)	51 (−8.5)	Saquinavir
28	ZINC01482077	24 (−9.7)	52 (−8.8)	70 (−9.6)	128 (−8.2)	Gliquidone
29 **	ZINC40164432	24 (−9.7)	77 (−8.7)	282 (−8.5)	215 (−8)	Lutein
30	ZINC53682927	24 (−9.7)	52 (−8.8)	60 (−9.7)	368 (−7.7)	NADH
31	ZINC21982951	24 (−9.7)	177 (−8.4)	70 (−9.6)	505 (−7.5)	Dasatinib
32	ZINC01493878	31 (−9.6)	52 (−8.8)	60 (−9.7)	8 (−8.9)	Sorafenib
33	ZINC18098320	31 (−9.6)	108 (−8.6)	24 (−10.3)	51 (−8.5)	Chlorhexidine
34	ZINC04214700	31 (−9.6)	23 (−9.2)	60 (−9.7)	168 (−8.1)	Paliperidone
35 **	ZINC08221225	31 (−9.6)	52 (−8.8)	70 (−9.6)	215 (−8)	Lutein
36	ZINC00537877	31 (−9.6)	177 (−8.4)	46 (−9.9)	19 (−8.8)	Ketanserin
37	ZINC03830629	31 (−9.6)	142 (−8.5)	554 (−7.9)	623 (−7.3)	Danazol
38	ZINC11678097	31 (−9.6)	77 (−8.7)	70 (−9.6)	433 (−7.6)	(7R,9R)-9-acetyl-7-[(2S,4R,5S,6S)-4-amino-5-hydroxy-6-methyl-tetrahydro pyran-2-yl]oxy-4,6,9,11-tetra
39	ZINC03097990	31 (−9.6)	218 (−8.3)	32 (−10.1)	51 (−8.5)	(2R,5S,6R)-7-keto-6-[[(2S)-2-[(2-keto-imidazolidine-1-carbonyl)amino]-2-phenyl-acetyl]amino]-3,3-dime
40	ZINC03817234	41 (−9.5)	77 (−8.7)	18 (−10.5)	24 (−8.7)	Maraviroc
41	ZINC00537791	41 (−9.5)	52 (−8.8)	8 (−10.7)	128 (−8.2)	Glimepiride
42	ZINC00968279	41 (−9.5)	142 (−8.5)	154 (−9)	128 (−8.2)	Troglitazone
43	ZINC00601274	41 (−9.5)	40 (−8.9)	37 (−10)	78 (−8.4)	Astemizole
44	ZINC03830631	41 (−9.5)	77 (−8.7)	51 (−9.8)	273 (−7.9)	Daunorubcin hydrochloride
45	ZINC03830557	41 (−9.5)	306 (−8)	121 (−9.2)	314 (−7.8)	Carmine
46	ZINC03830383	41 (−9.5)	11 (−9.4)	51 (−9.8)	51 (−8.5)	CCRIS 961; Carminomycin; Carubicin; Karminomycin; LS-86968; O-Demethyl-daunomycin
47 **	ZINC03830974	41 (−9.5)	23 (−9)	5 (−10.8)	51 (−8.5)	Itraconazole
48	ZINC03977985	41 (−9.5)	31 (−9)	353 (−8.3)	215 (−8)	Flumethasone pivalate
49	ZINC19796168	41 (−9.5)	450 (−7.8)	141 (−9.1)	168 (−8.1)	Sildenafil citrate
50	ZINC00601274	41 (−9.5)	31 (−9)	37 (−10)	78 (−8.4)	Astemizole

* Compounds that display the binding affinity > −9.5 kcal/mol with BmCALU-1. ** Structural and functional isomers: no. 4 and 5; no. 7 and 10; no. 8, 19, and 47; no. 11 and 12; no. 29 and 35.

## Supplementary Materials

Supplementary materials can be found at http://www.mdpi.com/1422-0067/19/11/3579/s1.
