# Supplementary material for: Novel Findings of Anti-Filarial Drug Target and Structure-Based Virtual Screening for Drug Discovery
Source: Int J Mol Sci. 2018 Nov 13;19(11):3579. doi: 10.3390/ijms19113579 (PMC6274684; doi:10.3390/ijms19113579)
Supplement: Supplementary file 1 [file ijms-19-03579-s001.pdf]

Supplementary Figure S1 and Table S1:

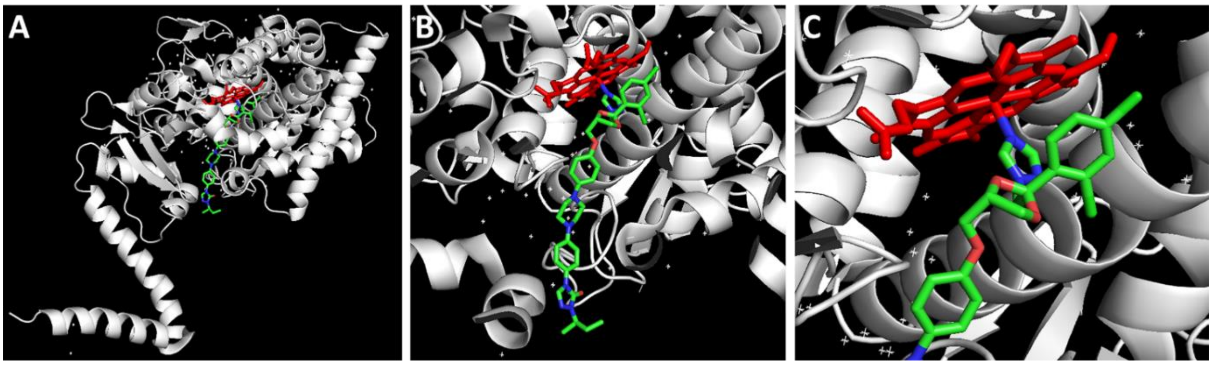

Figure S1. Docking mode of ITC to *Saccharomyces cerevisiae* lanosterol 14-α-demethylase (PDB accession no. 5EQB). Overview of binding (**A**) and detailed magnified images (**B**–**C**). *S. cerevisiae* lanosterol 14-α-demethylase is shown as gray. Active site heme is shown as red sticks. ITC carbon atoms are colored green, oxygen red and nitrogen blue.

Table S1. The top 10 threading templates used to build an individual model.

Rank *	Organism			
	**BmCALU-1**	**CeCALU-1**	**OvCALU-1**	**HsCALU-1**
1	2f33A	2f33A	2f33A	4p5wA
2	2be4A	4p5wA	4p5wA	4p5wA
3	lij5A	2be4A	2be4A	1ij5A
4	2be4A	1ij5A	2be4A	3q5iA
5	2q4uA	2be4A	2f33A	4il1A
6	1ij5A	1ij5A	1ij5A	1ij5A
7	4aqrA	1ij5A	2f33A	1ij5A
8	2q4uA	4aqrA	4aqrA	4aqrA
9	2f33A	2q4uA	2f33A	1ij5A
10	2be4A	2f33A	2f33A	1ij5A

* The top 10 alignments (in order of their ranking) are from the following threading programs: 1, MUSTER; 2, FFAS-3D; 3, SPARKS-X; 4, HHSEARCH2; 5, HHSEARCH I; 6, Neff-PPAS; 7, HHSEARCH; 8, pGenTHREADER; 9, wdPPAS; 10, cdPPAS.
